# Prevalence, clinical characteristics and management of low-renin hypertension: a real-world cohort study

**DOI:** 10.1038/s41371-026-01148-3

**Published:** 2026-05-12

**Authors:** Sonali S. Shah, Chrislyn Ng, Minn Wei Chow, Peter J. Fuller, Morag J. Young, Renata Libianto, Jun Yang

**Affiliations:** 1 Center for Endocrinology and Reproductive Health, Hudson Institute of Medical Research, Clayton, VIC Australia; 2 Department of Endocrinology, Monash Health, Clayton, VIC Australia; 3 Department of Molecular and Translational Science, Monash University, Clayton, VIC Australia; 4 Baker Heart and Diabetes Institute, Prahran, VIC Australia; 5 School of Translational Medicine, Monash University, Prahran, VIC Australia; 6 The Baker and University of Melbourne Department of Cardiometabolic Health and Disease, University of Melbourne, Vic, Australia

**Keywords:** Adrenal gland diseases, Hypertension

## Abstract

Emerging evidence suggests that patients with low-renin hypertension (LRH) benefit from targeted treatment with mineralocorticoid receptor antagonists (MRA). With broader screening for primary aldosteronism (PA), clinicians are increasingly identifying patients with low renin levels who do not meet current diagnostic criteria for PA. In the absence of specific management guidelines, these patients are often treated as having primary hypertension. This study aimed to assess the prevalence, clinical characteristics, and real-world management of LRH in a tertiary endocrine hypertension clinic. We conducted a retrospective audit of patients evaluated between 2016 and 2021. PA was diagnosed based on saline suppression test (SST) results. LRH and normal renin hypertension (NRH) were classified using a direct renin concentration below or above 10mU/L, respectively. Among 409 eligible patients, 82 (20%) had LRH, 221 (54%) had PA, and 106 (26%) had NRH. Compared to NRH, patients with LRH were older despite a similar duration of hypertension, more likely to be female, and non-Caucasian. They also had a lower 24-h urinary aldosterone excretion compared to both PA and NRH. Automated office blood pressure, urinary sodium, and potassium excretion were similar across groups. Only 25 patients with LRH (31%) were prescribed an MRA; others were prescribed conventional antihypertensive agents and/or discharged to their primary care physician. LRH was present in one-fifth of patients referred to for endocrine hypertension assessment. Management was inconsistent, underscoring the need for evidence-based diagnostic and treatment strategies for this under-recognized subtype of hypertension.

## Introduction

Hypertension is the leading modifiable risk factor for cardiovascular morbidity and mortality globally. Primary aldosteronism (PA), characterized by inappropriate aldosterone production relative to an individual’s volume and sodium status, is increasingly recognized as an important contributor to hypertension, affecting up to 13% of people with hypertension in primary care and up to 30% in referral centers [1].

Many societies, including the Endocrine Society and European Society of Cardiology, advocate screening all hypertensive individuals with renin and aldosterone measurements to facilitate early detection of PA and initiation of targeted treatment, to reduce blood pressure and cardiovascular risk [2–4].

The diagnosis of PA typically relies on aldosterone suppression testing using intravenous saline, oral salt loading, or angiotensin-converting enzyme inhibitors, with a fixed aldosterone threshold to assess the adequacy of suppression [4]. However, these dichotomous criteria will exclude patients with low-renin hypertension (LRH), and may exclude those with mild autonomous aldosterone production, who do not meet the current diagnostic criteria for PA but may also benefit from targeted treatment with mineralocorticoid receptor (MR) antagonists (MRA) [5]. A meta-analysis of randomized clinical trials that included patients with LRH (PA excluded) showed that MRA were superior in lowering blood pressure compared to angiotensin converting enzyme inhibitors and angiotensin receptor blockers, and were similar in efficacy to epithelial sodium channel inhibitors (ENaCi) [6]. These findings suggest that many individuals with hypertension, low renin, and an aldosterone concentration below the diagnostic cutoff for PA may still benefit from MR-targeted therapy rather than being managed as having primary hypertension. A likely consequence of expanded PA screening is increased detection of LRH, providing an opportunity for earlier initiation of effective treatment.

Real-world data on how these individuals are currently managed in clinical practice are lacking. We therefore conducted this cohort study to: 1) quantify the proportion of patients referred to an Endocrine Hypertension clinic at a tertiary hospital who have LRH, 2) describe their clinical characteristics, and 3) assess their management in the absence of specific guidelines.

## Methods

### Study design

We performed a retrospective analysis of consecutive patients seen at the Endocrine Hypertension Clinic at Monash Health between its inception in 2016 and 2021. Since opening, the clinic has expanded its capacity to accommodate increasing referral demand over the audit period. Monash Health, the largest tertiary public health service in Victoria, Australia, receives referrals from primary care physicians and other specialists and serves a culturally and socioeconomically diverse population. Patients were included if they attended the clinic at least once and had both aldosterone and renin levels measured while not taking antihypertensive medications known to interfere with the renin-angiotensin-aldosterone system.

### Saline suppression test and definitions for the subtype of hypertension

At Monash Health, patients with a positive screening ARR defined as >70 pmol/mU (25 ng/mU), measured off interfering antihypertensive medications, were referred for confirmatory testing with a saline suppression test (SST) (2 L of 0.9% saline over 4 h) [4]. Inadequate aldosterone suppression, consistent with a diagnosis of PA, was defined as a post-saline aldosterone concentration >140pmol/L (5.1 ng/dL) supine or >170pmol/L (6.1 ng/dL) seated [4, 7]. Patients who did not meet diagnostic criteria for PA were further classified based on their direct renin concentration (DRC): LRH was defined by a DRC <10mU/L, and normal-renin hypertension (NRH) was defined by a DRC ≥10mU/L.

For patients classified as having LRH, we collected data on their clinical management, including whether MRA was commenced and whether their care was referred to a primary care physician.

### Clinical parameters

Ethnicity, cardio-metabolic co-morbidities, and antihypertensive medications were self-reported. Antihypertensives were quantified using the World Health Organization’s Defined Daily Dose index. Automated office blood pressure was measured in the seated position in triplicate, and the mean of the 2^nd^ and 3^rd^ measurement was calculated at baseline and 12 months from treatment initiation. Body mass index was calculated at the initial clinic visit as follows: weight in kilograms divided by height in meters squared.

### Biochemistry

Plasma aldosterone and renin were measured at seven accredited laboratories, which all utilized the DiaSorin LIAISON® chemiluminescent immunoassays on the Liaison XL analyzer (DiaSorin, Saluggia, Italy). Clinic patients are routinely advised to have their pathology tests done in a seated position, approximately two hours after waking, with no change in their dietary salt intake.

For the aldosterone assay, the quoted between-run analytical coefficients of variation were 9.5% at 188 pmol/L and 5.6% at 798 pmol/L (1 ng/dL = 277 pmol/L). The DRC was reported in mU/L; the quoted between-run analytical coefficients of variation were 10.0% at 5.1 mU/L and 4.3% at 82.4 mU/L. Although there is no direct conversion factor available between DRC and plasma renin activity, 1 mU/L approximately is equal to 0.1 ng/ml/hour.

Serum lipids, glucose, insulin, creatinine, and electrolytes (sodium, potassium) were measured using standard automated assays.

### Urine collection

Twenty-four-hour urine aldosterone, sodium, and potassium excretion were measured from a complete 24-h urine collection (acidified for preservation). Aldosterone was quantified by immunoassay.

### Statistical methods

Data distribution was assessed using the Kolmogorov–Smirnov test and visual inspection of boxplots. Continuous variables were summarized as medians with interquartile ranges (25^th^, 75^th^ percentiles). Group comparisons were performed using the Kruskal–Wallis test for continuous variables and the Chi-square test for categorical variables. A p-value of <0.05 was considered statistically significant. Post hoc comparisons were performed with a Bonferroni adjustment. All statistical analyses were conducted using IBM SPSS Statistics, version 28 (Armonk, NY, USA).

### Ethics approval

This clinical audit was conducted in accordance with the NHMRC National Statement on Ethical Conduct in Human Research (2025) and the Declaration of Helsinki. It was exempt from Human Research Ethics Committee review, and all data were de-identified to maintain patient confidentiality.

### Reporting

Findings are reported following the Strengthening the Reporting of Observational studies in Epidemiology (STROBE) framework.

## Results

### Proportion of clinic patients with LRH

**Fig. 1:**
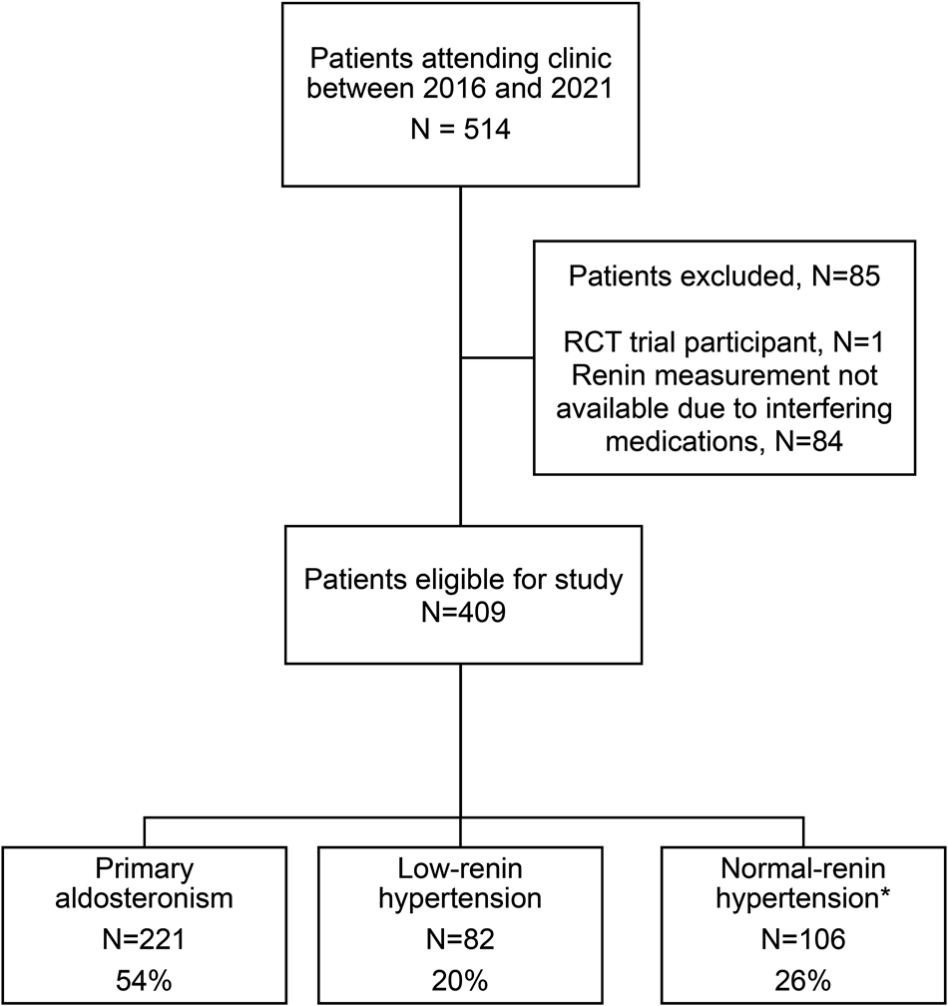
The flow chart shows the patients eligible for the study and the reasons for exclusion. The proportion of patients with primary aldosteronism, low-renin hypertension and normal-renin hypertension in the clinic is shown in percentages. N: number. *n = 13 with direct renin concentration >46.1mU/L.

**Fig. 2:**
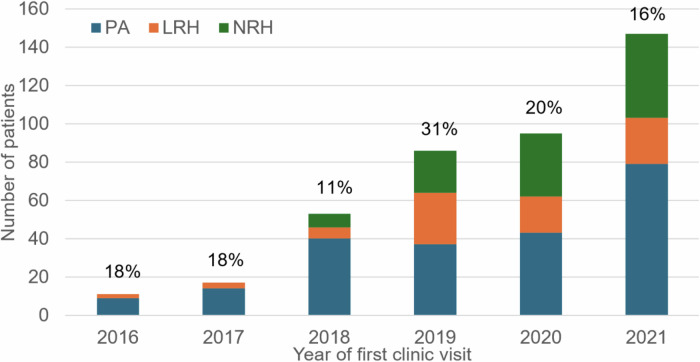
Annual clinic attendance and percentage of patients with low‑renin hypertension (2016–2021). The figure displays the number of patients seen in the endocrine hypertension clinic each year from 2016 to 2021, along with the proportion diagnosed with low‑renin hypertension shown in percentages. PA primary aldosteronism, LRH low-renin hypertension, NRH normal-renin hypertension.

A total of 514 patients were seen at the Endocrine Hypertension Clinic between 2016 and 2021. Eighty-four patients had renin and aldosterone measured on interfering medications and were therefore excluded. One patient entered a randomized trial that is currently underway and was also excluded [8]. Out of the 409 eligible patients, 82 (20%) were classified as having LRH, 221 (54%) had PA, and 106 (26%) had NRH (Fig. 1). The proportion of patients seen with LRH per year varied from 11% to 31% (Fig. 2).

### Saline suppression tests

Among those who underwent the seated SST (n = 172), the pre-saline serum potassium was lower in patients with PA compared to NRH (4.0 vs. 4.4 mmol/L, p < 0.05), but similar to that of patients with LRH (4.1 mmol/L) (Table 1). The median pre-saline aldosterone concentration was lower in patients with LRH compared to PA (292 pmol/L (10.5 ng/dL) vs. 464 pmol/L (16.7 ng/dL), p < 0.001) but similar to values for NRH (370 pmol/L). As expected, post-SST aldosterone concentration was lower in patients with LRH compared to PA (143 pmol/L (5.2 ng/dL) vs. 257 pmol/L (9.3 ng/dL), p < 0.001) but similar to patients with NRH (142 pmol/L (5.1 ng/dL)). A similar pattern was seen in patients who underwent a supine SST (n = 85) (Table 1).

**Table 1:** Baseline characteristics of patients.

	Diagnosis			Overall p-value
	LRH (N = 82)	NRH (N = 106)	PA (N = 221)	
Age at study entry (years)	55 (45, 65)	46 (36, 58) *†	53 (44, 59)	<0.001
Sex N (%) female	54 (66%)‡	51 (48%)	121 (55%)	0.051
Duration of hypertension (years)	2 (0,10)	4 (1,11)	4 (1,11)	0.172
*Ethnicity? N (%)*				
Caucasian	46 (61%)	72 (77%)§‡	129 (60%)	0.152
East Asian	5 (7%)	5 (5%)	13 (6%)	
South Asian	6 (8%)	7 (8%)	17 (8%)	
African	7 (9%)	4 (4%)	15 (7%)	
Middle Eastern	3 (4%)	1 (1%)	16 (8%)	
Polynesian	1 (1%)	2 (2%)	11 (5%)	
Other	7 (9%)	2 (2%)	13 6%)	
Aldosterone (pmol/L)	314 (219, 500)	368 (269, 516)	452 (344, 641)§||	<0.001
Renin (mU/L)	3.1 (2.0, 6.2)	20.2 (13.5, 29.4)*†	3.7 (2.0, 6.1)	<0.001
ARR (pmol/mU)	105 (52, 201)	20 (12, 27)*†	123 (81, 213)	<0.001
eGFR (mL/min)	90 (78, 90)	90 (77, 90)	90 (82, 90)	0.853
Sodium (mmol/L)	141 (139, 142)	141 (139, 142)	141 (139, 142)	0.679
Potassium (mmol/L)	4.2 (4.0, 4.5)	4.3 (4.0, 4.6)	4.1 (3.8, 4.3)†#	<0.001
Bicarbonate (mmol/L)	27 (25, 29)	27 (26, 28)	27 (26, 29)	0.131
Creatinine (umol/L)	65 (55, 81)*	79 (65, 88)	70 (61, 84)	<0.001
*Urine excretion*	*N* = *42*	*N* = *64*	*N* = *113*	
Spot urine Albumin: creatinine ratio (mg/mmol)	1.1 (0.8, 2.9)	1.2 (0.6, 5.6)	1.6 (0.9, 3.6)	0.525
Sodium excretion (mmol/day)	129 (95, 201)	141 (98, 200)	130 (97, 207)	0.896
Potassium excretion (mmol/day)	61 (48, 84)	73 (54, 90)	72 (57, 98)	0.162
Aldosterone excretion (nmol/day)	22 (14, 28)‡||	29 (21, 43)	33 (22, 46)	<0.001
*Supine saline suppression test*	*N* = *3*		*N* = *82*	
Pre: Potassium (mmol/L)	4.2 (3.9,4.6)		3.9 (3.8 4.2)	0.378
Pre: Aldosterone (pmol/L)	170 (160, 211)		408 (282, 538)	0.012
Pre: Cortisol (nmol/L)	181 (175, 223)		254 (190, 326)	0.215
Pre: Renin (mU/L)	2.1 (1.0, 2.6)		2.9 (1.3, 5.7)	0.385
Pre: ARR (supine, pmol/mU)	76 (55, 164)		148 (71, 275)	0.552
Post: Potassium (mmol/L)	4.2 (4.2, 4.4)		3.8 (3.6, 4.1)	0.035
Post: Aldosterone (pmol/L)	107 (100, 112)		230 (171, 333)	0.004
Post: Cortisol (nmol/L)	105 (89, 143)		166 (129, 229)	0.156
Post: Renin (mU/L)	1.3 (0.5, 2.1)		1.8 (0.8, 3.0)	0.541
Post: ARR (pmol/mU)	64 (38, 89)		142 (79, 264)	0.118
*Seated saline suppression test*	*N* = *31*	*N* = *6*	*N* = *135*	
Pre: Potassium (mmol/L)	4.1 (3.9, 4.2)	4.4 (4.2, 4.5)§	4.0 (3.8, 4.2)	0.021
Pre: Aldosterone (pmol/L)	293 (239, 424)||	370 (290, 480)	464 (351, 668)	<0.001
Pre: Cortisol (nmol/L)	226 (176, 347)	301 (218, 389)	282 (221, 338)	0.183
Pre: Renin (mU/L)	3.5 (1.8, 6.7)	13.5 (9.1, 25.0)‡§	5.2 (2.2, 9.6)	0.005
Pre: ARR (pmol/mU)	75 (48, 167)	29 (16, 41) ‡§	87 (49, 155)	0.004
Post: Aldosterone (pmol/L)	143 (123, 164)	142 (117, 162)	257 (220, 370)†||	<0.001
Post: Potassium (mmol/L)	3.9 (3.8, 4.2)	4.4 (4.0, 4.6)	3.9 (3.7, 4.2)	0.062
Post: Cortisol (nmol/L)	129 (100, 185)	159 (121, 234)	173 (128, 218)	0.128
Post: Renin (mU/L)	1.9 (1.2, 3.9)‡	6.5 (5.0, 12.8)	3.0 (1.8, 5.7)	0.012
Post: ARR (pmol/mU)	75 (39, 110)	21 (13, 27)‡§	78 (48, 142)	0.001

^*^NRH vs. LRH p < 0.001, †NRH vs. PA p < 0.001, ‡NRH vs. LRH p < 0.05, § NRH vs PA p < 0.05, ||LRH vs. PA p < 0.001 and #LRH vs. PA p < 0.05.

*ARR* aldosterone-to-renin ratio, *eGFR* estimated glomerular filtration rate, *LRH* low-renin hypertension, *N* number, *NRH* normal-renin hypertension, *PA* primary aldosteronism.

### Clinical and biochemical characteristics

Patients with NRH were younger compared to those with PA and LRH (46 years vs. 53 years and 55 years, p < 0.001). However, the duration of hypertension was similar across the three groups (NRH and PA: 4 years, and LRH: 2 years, p = 0.172) (Table 1).

In the LRH group, a higher proportion were female compared to the NRH group (66% vs. 48%, p < 0.05), and fewer were Caucasian (61% vs. 77%, p < 0.05). The remainder of patients with LRH were East Asian (7%), South Asian (8%), African (9%), Middle Eastern (4%), or Polynesian (1%).

In patients with PA, median serum potassium was somewhat lower compared to those with NRH and LRH (4.1 mmol/L vs. 4.3 and 4.2 mmol/L, p < 0.05), but urinary potassium excretion was comparable across groups: 72 mmol/day vs. 73 mmol/day and 61 mmol/day, p = 0.162). Despite a similar median aldosterone concentration for LRH and NRH, the 24-h urinary aldosterone excretion was lower in LRH compared to NRH (22 nmol/day vs. 29 nmol/day, p < 0.05). Median serum sodium and urinary sodium excretion were also similar across all groups.

Serum creatine was lower in those with LRH compared to NRH (65 umol/L vs. 79 umol/L, p < 0.001) but similar to PA (70 umol/L). When stratified by sex, this difference persisted only in females (p = 0.009), not in males (p = 1.000) (Supplementary Figure 1). Spot urine albumin-creatinine ratio was similar across groups (LRH: 1.1, NRH: 1.2, and PA: 1.6 mg/mmol, p = 0.525).

### Baseline blood pressure, cardiometabolic characteristics, and antihypertensive use

Median automated office blood pressure in patients with LRH (SBP: 140 mmHg and DBP: 89 mmHg) was similar to that of those with NRH (Table 2). However, patients with NRH had a lower SBP and DBP compared to those with PA (p < 0.001). Anti-hypertensive medication use, expressed as WHO-defined daily dose (DDD), was similar across all groups (LRH: 1.3, NRH: 1.1, and PA: 1.1).

**Table 2:** Baseline blood pressure, antihypertensive use, and cardiometabolic characteristics.

	Diagnosis			Overall p-value
	LRH N = 82	NRH N = 106	PA N = 221	
Stroke N (%)	6 (7%)	9 (9%)	8 (4%)	0.153
Ischemic heart disease N (%)	3 (4%)	6 (6%)	3 (1%)	0.089
Arrhythmia N (%)	5 (6%)	6 (6%)	8 (4%)	0.559
Chronic kidney disease N (%)	3 (4%)	9 (9%)	13 (6%)	0.382
Dyslipidemia N (%)	30 (37%)	32 (30%)	67 (30%)	0.546
Diabetes N (%)	11 (13%)	15 (14%)	28 (13%)	0.932
Obstructive sleep apnea N (%)	10 (12%)	12 (11%)	32 (15%)	0.700
Heart failure N (%)	2 (2%)	0 (0%)	2 (1%)	0.239
Number of current antihypertensives used	1 (1, 2)	1 (1, 2)	1 (1, 2)	0.860
Defined daily dose of baseline antihypertensives (other than MRA)	1.0 (0.2, 3.0)	1.1 (0.4, 3.0)	1.0 (0.0, 3.0)	0.801
Defined daily dose of MRA at baseline	0.7 (0.3, 1.3)*	0.0 (0.0, 0.3)	0.0 (0.0, 0.7)	0.028
Total defined daily dose of antihypertensives at baseline	1.3 (0.4,3.0)	1.1 (0.4, 3.0)	1.1 (0.3, 3.1)	0.866
Systolic blood pressure (mmHg)	140 (124, 155)	135 (128, 148)†	145 (133, 158)	<0.001
Diastolic blood pressure (mmHg)	89 (82, 95)	85 (78, 95)†	91 (84, 99)	<0.001
BMI (kg/m2)	28.7 (25.0, 33.9)	29.8 (26.4, 36.4)	28.8 (25.4, 33.0)	0.083
*Metabolic profile*	*N* = *52*	*N* = *78*	*N* = *99*	
Glucose (fasting, mmol/L)	5.2 (5.0, 6.0)	5.5 (5.0, 6.2)	5.4 (5.0, 5.9)	0.748
Insulin (fasting, mU/L)	11.0 (6.7, 17.6)	13.0 (7.0, 21.6)‡	9.0 (5.0, 12.4)	0.048
Total cholesterol (fasting, mmol/L)	5.2 (4.5, 6.1)	4.8 (4.0, 5.8)	5.1 (4.7, 5.8)	0.128
LDL (fasting, mmol/L)	3.2 (2.6, 3.8)	2.7 (2.2, 3.6)‡	3.1 (2.6, 3.8)	0.016
HDL (fasting, mmol/L)	1.4 (1.2, 1.7)	1.3 (1.1, 1.6)	1.3 (0.9, 1.7)	0.504
Triglyceride (fasting, mmol/L)	1.4 (1.1, 1.8)	1.3 (0.9, 2.0)	1.3 (0.9, 1.6)	0.508

*BMI* body mass index, *HDL* high-density lipoprotein; low-density lipoprotein, *LRH* low-renin hypertension, *MRA* mineralocorticoid receptor antagonists, *N* number, *NRH* normal-renin hypertension; PA: primary aldosteronism.

*LRH vs. NRH p < 0.05, †NRH vs PA p < 0.001 and ‡ NRH vs PA p < 0.05.

Fasting insulin and low-density lipoprotein (LDL) cholesterol levels were similar in patients with LRH (11.0 mU/L and 3.2 mmol/L, respectively) when compared to those with NRH and PA. Fasting total cholesterol, high-density lipoprotein (HDL), triglyceride, glucose, and body mass index were similar for all groups.

Among patients with LRH, 37% had concomitant dyslipidemia. Smaller proportions of patients with LRH had diabetes (13%), obstructive sleep apnea (12%), stroke (7%), ischemic heart disease (4%), heart failure (3%), arrhythmia (6%), and chronic kidney disease (4%). The prevalence of co-morbidities was comparable across the groups.

### Management of patients with low-renin hypertension

Of the 82 patients with LRH, 25 (31%) were prescribed MRA, 4 (5%) angiotensin converting inhibitors or angiotensin II receptor blockers, 2 (2%) diuretics (non-potassium sparing), 5 (6%) beta blockers, 10 (11%) calcium channel blockers, 3 (4%) alpha-1 blockers (prazosin), 5 (6%) alpha-2/imidazoline receptor agonist (moxonidine) and 1 (1%) direct vasodilator (hydralazine). The remainder were not commenced on anti-hypertensives. Approximately half of the patients (n = 40, 49%) were discharged to a primary physician for management of their hypertension.

Median 12-month automated office blood pressure was 130/79 mmHg in the LRH group (n = 33). There were no significant differences between groups in terms of 12-month blood pressure (both absolute and change from baseline) or DDD of antihypertensives (Table 3). Due to small sample sizes, blood pressure outcomes within the LRH group were not compared across different antihypertensive drug classes.

**Table 3:** Automated office blood pressure and anti-hypertensive use at 12 months follow-up.

	Diagnosis			Overall p-value
	LRH	NRH	PA	
*Automated office blood pressure*	*N* = *33*	*N* = *7*	*N* = *183*	
SBP (mmHg)	130 (123, 134)	125 (115, 136)	130 (123, 137)	0.530
DBP (mmHg)	79 (76, 86)	85 (75, 90)	85 (78, 90)	0.056
Change in SBP (mmHg)	-13 (-26, -5)	-19 (-27, 5)	-17 (-27, -1)	0.780
Change in DBP (mmHg)	-13 (-18, -4)	-10 (-11, -4)	-7 (-15, 1)	0.090
*Anti-hypertensive use*	*N* = *25*	*N* = *3*	*N* = *140*	
DDD of antihypertensives (other than MRA)	0.3 (0.0, 2.9)	N/A	0.0 (0.0, 1.0)	0.119
DDD of MRA	1.3 (0.7, 1.9)	N/A	1.3 (0.7, 1.3)	0.740
Total DDD of antihypertensives	1.8 (0.7, 4.7)	N/A	1.4 (1.0, 2.7)	0.650

*DBP* diastolic blood pressure, *DDD* defined daily dose, *LRH* low-renin hypertension, *MRA* mineralocorticoid receptor antagonists, *N* number, *NRH* normal-renin hypertension, *PA* primary aldosteronism, *SBP* systolic blood pressure.

## Discussion

This study demonstrates that a significant proportion of patients referred to a tertiary endocrine hypertension clinic have low renin levels but do not meet the current biochemical criteria for PA. Concerningly, only one-third of these patients received targeted treatment despite growing evidence supporting the efficacy of MRA or ENaCi for treating LRH [6, 9–12]. The variability in clinical management likely reflects the lack of clear diagnostic and therapeutic guidelines for LRH, resulting in these patients often being managed similarly to those with primary hypertension.

In our cohort of patients seen at a specialized clinic, 20% were found to have LRH. This prevalence is comparable to findings in other populations. In a large study of 1267 hypertensive patients referred to a specialized center in China, 26% of patients had LRH, defined as a plasma renin activity <1 ng/ml/hour ( ~ DRC concentration ≤10 mU/L) and not meeting the criteria for PA [13]. This is consistent when dynamic testing is used to define LRH; in the HyperPath Consortium, a multisite study aimed at investigating the pathophysiology of hypertension in tertiary centers in USA and France, 19% of participants had LRH, defined by a stimulated plasma renin activity ≤2.4 ng/ml/hour ( ~ DRC ≤ 24 mU/L) after low salt diet and upright posture [14]. Interestingly, the prevalence of LRH is similar even in primary care, suggesting a more widespread and under-recognized clinical issue. In a prospective study of treatment-naïve hypertensive patients in primary care, 26% were found to have LRH [15]. This highlights the emerging challenge of managing a substantial subgroup of hypertensive patients for whom standard treatment guidelines may not apply.

The clinical and biochemical characteristics of patients with LRH in this study are similar to those reported previously: older age, a higher proportion of females, lower representation of Caucasian people, and lower aldosterone concentrations compared to those with NRH [15, 16]. Although not statistically significant, in this study, there was a trend toward higher proportions of patients identifying as Asian or African in the LRH group (24% vs. 17%), warranting further exploration in larger cohorts. These characteristics may be clues to the underlying pathophysiology of LRH, such as early/emerging aldosterone autonomy due to an age-related accumulation of aldosterone-producing adrenal cell clusters, or increased salt sensitivity in females and individuals of African-Caribbean or Asian background [17, 18]. Although a common cause of low renin and low aldosterone is high dietary salt intake, median sodium excretion, which is reflective of sodium intake, was similar for all groups. However, interpretation of this is limited as a 24-hour measurement provides a snapshot of current sodium intake and does not represent long-term exposure. Additionally, the power to detect differences may be reduced due to missing data (approximately 50% in each group did not have a 24-hour urine collection) and the potential inaccuracy of unsupervised outpatient collections.

An interesting finding was that female patients with LRH had lower serum creatinine compared with those with NRH. The underlying reason is unclear, as there were no differences in body mass index or reported cardiorenal disease between groups. Potential explanations include lower muscle mass due to differences in body composition despite similar body mass index, particularly given the older age of the LRH group. Alternatively, this finding may reflect glomerular hyperfiltration in LRH, analogous to that observed in PA, whereby intravascular volume expansion and alterations in glomerular hemodynamics (afferent arteriolar vasodilation and/or efferent arteriolar vasoconstriction) lead to artificially lower serum creatinine and inflated eGFR [19, 20]. No differences in serum creatinine were observed among male patients. These finding warrants further investigation in larger cohorts incorporating cystatin C measurements.

The strengths of this study include its real-world evaluation of a large, well-characterized cohort from a tertiary endocrine hypertension clinic, capturing detailed clinical, biochemical, and management data.

However, this study has several limitations. First, the classification of LRH and NRH was based on a dichotomous DRC threshold of 10 mU/L, selected based on prior evidence showing maximal blood pressure-lowering response to MRA in patients with renin concentration <9.4 mU/L [21]. However, this binary threshold may oversimplify a continuum, as demonstrated by the PATHWAY-2 trial. In this randomized clinic trial of add-on therapy for participants with resistant hypertension, an inverse relationship between renin concentration and blood pressure response to spironolactone was observed [10]. Second, not all patients in the clinic underwent medication adjustment or SST, often due to comorbidities or patient preference. There was also a change in the local SST protocol from a supine to a seated position, with corresponding post-SST aldosterone thresholds of 140 and 170 pmol/L, respectively, to improve the sensitivity of PA diagnosis. However, this change may have occurred at the expense of specificity, and led some patients with mild PA to be diagnosed as having LRH, thus over-estimating the prevalence of LRH [22]. These factors may have led to over- or under-estimation of LRH prevalence. Third, due to extensive engagement with primary care practitioners within the referral catchment and increased uptake of PA screening [23], a relatively high proportion of patients in this cohort had a milder PA phenotype. Consequently, these findings may not be generalisable to an unselected hypertensive population or to other tertiary referral centres without similar primary care screening practices. Finally, missing data on urinary excretion may have limited our power to detect differences in urinary sodium excretion between groups and differences in treatment efficacy between hypertension groups and between drug classes could not be adequately assessed, as only a small number of patients with LRH and NRH were followed up in the clinic. Future work should evaluate whether some or all patients with LRH experience improvements in blood pressure, potassium, and renin profiles when treated with MRA/ENaCi, and whether this translates into reduced cardiovascular risk compared to those managed with conventional antihypertensive regimens.

This retrospective study highlights that a substantial proportion of patients referred to a tertiary hypertension clinic have LRH, which falls outside current diagnostic thresholds for PA. Clinical management in this group varies widely, reflecting the lack of specific guidelines. As more hypertension guidelines advocate for PA screening [2–4], the number of people identified to have hypertension and low renin is expected to rise. With increasing detection of LRH and emerging evidence for using MRA or ENaCi to treat LRH, there is an urgent need for multicenter prospective studies to evaluate blood pressure and cardiovascular outcomes in patients with LRH treated with MR- and ENaC-targeted therapies, compared with conventional antihypertensives, to better inform future hypertension guidelines.

## Summary

### What is known about this topic?

- Renin and aldosterone measurements are increasingly being used in the diagnostic work-up of individuals with hypertension, enabling earlier detection of primary aldosteronism (PA), which has well-established treatment pathways.
- However, an anticipated by-product of this expanded screening is the growing recognition of people with low-renin hypertension who have normal or subthreshold aldosterone levels that do not meet diagnostic criteria for PA.
- A current gap in hypertension guidelines is that low-renin hypertension is not specifically addressed, despite growing evidence that people with low-renin (even without PA) may benefit from mineralocorticoid receptor/epithelial sodium channel–targeted therapy.

### What is new?

- This study provides detailed real-world evaluations of low-renin hypertension in a tertiary endocrine hypertension clinic, identifying that 20% of patients being investigated for PA fall into this category.
- This study reveals varied management of low-renin hypertension, with only one-third receiving mineralocorticoid receptor antagonists, despite emerging evidence supporting the use of this targeted treatment.
- It highlights the need for further research in the optimal management of low-renin hypertension to inform future clinical practice guidelines.

## Supplementary information

Supplemental material

## Data Availability

Data is available from the corresponding author upon reasonable request.
