# Supplementary material for: Prevalence, clinical characteristics and management of low-renin hypertension: a real-world cohort study
Source: J Hum Hypertens. 2026 May 12;40(7):541–7. doi: 10.1038/s41371-026-01148-3 (PMC13345966; doi:10.1038/s41371-026-01148-3)
Supplement: Supplementary file 1 — Supplemental material [file 41371_2026_1148_MOESM1_ESM.docx]

**Supplementary data**

Table S1: Missing data.

|  | Diagnosis | Cases | | | | | |
| --- | --- | --- | --- | --- | --- | --- | --- |
|  |  | Valid | | Missing | | Total | |
|  |  | N | Percent | N | Percent | N | Percent |
| Age at study entry (years) | LRH | 82 | 100.0% | 0 | 0.0% | 82 | 100.0% |
|  | NRH | 106 | 100.0% | 0 | 0.0% | 106 | 100.0% |
|  | PA | 221 | 100.0% | 0 | 0.0% | 221 | 100.0% |
| Sex | LRH | 82 | 100.0% | 0 | 0.0% | 82 | 100.0% |
|  | NRH | 106 | 100.0% | 0 | 0.0% | 106 | 100.0% |
|  | PA | 221 | 100.0% | 0 | 0.0% | 221 | 100.0% |
| What is your ethnicity? | LRH | 75 | 91.5% | 7 | 8.5% | 82 | 100.0% |
|  | NRH | 93 | 87.7% | 13 | 12.3% | 106 | 100.0% |
|  | PA | 214 | 96.8% | 7 | 3.2% | 221 | 100.0% |
| Duration of hypertension (years) | LRH | 80 | 97.6% | 2 | 2.4% | 82 | 100.0% |
|  | NRH | 102 | 96.2% | 4 | 3.8% | 106 | 100.0% |
|  | PA | 216 | 97.7% | 5 | 2.3% | 221 | 100.0% |
| Stroke | LRH | 82 | 100.0% | 0 | 0.0% | 82 | 100.0% |
|  | NRH | 106 | 100.0% | 0 | 0.0% | 106 | 100.0% |
|  | PA | 221 | 100.0% | 0 | 0.0% | 221 | 100.0% |
| Ischemic heart disease | LRH | 82 | 100.0% | 0 | 0.0% | 82 | 100.0% |
|  | NRH | 106 | 100.0% | 0 | 0.0% | 106 | 100.0% |
|  | PA | 221 | 100.0% | 0 | 0.0% | 221 | 100.0% |
| Arrhythmia | LRH | 82 | 100.0% | 0 | 0.0% | 82 | 100.0% |
|  | NRH | 106 | 100.0% | 0 | 0.0% | 106 | 100.0% |
|  | PA | 221 | 100.0% | 0 | 0.0% | 221 | 100.0% |
| Chronic kidney disease | LRH | 82 | 100.0% | 0 | 0.0% | 82 | 100.0% |
|  | NRH | 106 | 100.0% | 0 | 0.0% | 106 | 100.0% |
|  | PA | 221 | 100.0% | 0 | 0.0% | 221 | 100.0% |
| Dyslipidemia | LRH | 82 | 100.0% | 0 | 0.0% | 82 | 100.0% |
|  | NRH | 106 | 100.0% | 0 | 0.0% | 106 | 100.0% |
|  | PA | 221 | 100.0% | 0 | 0.0% | 221 | 100.0% |
| Diabetes | LRH | 82 | 100.0% | 0 | 0.0% | 82 | 100.0% |
|  | NRH | 106 | 100.0% | 0 | 0.0% | 106 | 100.0% |
|  | PA | 221 | 100.0% | 0 | 0.0% | 221 | 100.0% |
| Obstructive sleep apnea | LRH | 82 | 100.0% | 0 | 0.0% | 82 | 100.0% |
|  | NRH | 106 | 100.0% | 0 | 0.0% | 106 | 100.0% |
|  | PA | 221 | 100.0% | 0 | 0.0% | 221 | 100.0% |
| Heart failure | LRH | 82 | 100.0% | 0 | 0.0% | 82 | 100.0% |
|  | NRH | 106 | 100.0% | 0 | 0.0% | 106 | 100.0% |
|  | PA | 221 | 100.0% | 0 | 0.0% | 221 | 100.0% |
| Number of current antihypertensives used | LRH | 82 | 100.0% | 0 | 0.0% | 82 | 100.0% |
|  | NRH | 106 | 100.0% | 0 | 0.0% | 106 | 100.0% |
|  | PA | 219 | 99.1% | 2 | 0.9% | 221 | 100.0% |
| Defined daily dose of baseline antihypertensives (other than MRA) | LRH | 82 | 100.0% | 0 | 0.0% | 82 | 100.0% |
|  | NRH | 104 | 98.1% | 2 | 1.9% | 106 | 100.0% |
|  | PA | 219 | 99.1% | 2 | 0.9% | 221 | 100.0% |
| Defined daily dose of MRA at baseline | LRH | 15 | 18.3% | 67 | 81.7% | 82 | 100.0% |
|  | NRH | 8 | 7.5% | 98 | 92.5% | 106 | 100.0% |
|  | PA | 48 | 21.7% | 173 | 78.3% | 221 | 100.0% |
| Total defined daily dose of antihypertensives at baseline | LRH | 82 | 100.0% | 0 | 0.0% | 82 | 100.0% |
|  | NRH | 104 | 98.1% | 2 | 1.9% | 106 | 100.0% |
|  | PA | 219 | 99.1% | 2 | 0.9% | 221 | 100.0% |
|  | PA | 217 | 98.2% | 4 | 1.8% | 221 | 100.0% |
| What were the baseline antihypertensive medications? (choice=Mineralocorticoid receptor antagonist) | LRH | 82 | 100.0% | 0 | 0.0% | 82 | 100.0% |
|  | NRH | 106 | 100.0% | 0 | 0.0% | 106 | 100.0% |
|  | PA | 221 | 100.0% | 0 | 0.0% | 221 | 100.0% |
| What were the baseline antihypertensive medications? (choice=Diuretic) | LRH | 82 | 100.0% | 0 | 0.0% | 82 | 100.0% |
|  | NRH | 106 | 100.0% | 0 | 0.0% | 106 | 100.0% |
|  | PA | 221 | 100.0% | 0 | 0.0% | 221 | 100.0% |
| What were the baseline antihypertensive medications? (choice=ACE inhibitor / ARB) | LRH | 82 | 100.0% | 0 | 0.0% | 82 | 100.0% |
|  | NRH | 106 | 100.0% | 0 | 0.0% | 106 | 100.0% |
|  | PA | 221 | 100.0% | 0 | 0.0% | 221 | 100.0% |
| What were the baseline antihypertensive medications? (choice=Beta blocker) | LRH | 82 | 100.0% | 0 | 0.0% | 82 | 100.0% |
|  | NRH | 106 | 100.0% | 0 | 0.0% | 106 | 100.0% |
|  | PA | 221 | 100.0% | 0 | 0.0% | 221 | 100.0% |
| What were the baseline antihypertensive medications? (choice=Dihydropyridine calcium channel blocker) | LRH | 82 | 100.0% | 0 | 0.0% | 82 | 100.0% |
|  | NRH | 106 | 100.0% | 0 | 0.0% | 106 | 100.0% |
|  | PA | 221 | 100.0% | 0 | 0.0% | 221 | 100.0% |
| What were the baseline antihypertensive medications? (choice=Non-dihydropyridine calcium channel blocker) | LRH | 82 | 100.0% | 0 | 0.0% | 82 | 100.0% |
|  | NRH | 106 | 100.0% | 0 | 0.0% | 106 | 100.0% |
|  | PA | 221 | 100.0% | 0 | 0.0% | 221 | 100.0% |
| What were the baseline antihypertensive medications? (choice=Moxonidine) | LRH | 82 | 100.0% | 0 | 0.0% | 82 | 100.0% |
|  | NRH | 106 | 100.0% | 0 | 0.0% | 106 | 100.0% |
|  | PA | 221 | 100.0% | 0 | 0.0% | 221 | 100.0% |
| What were the baseline antihypertensive medications? (choice=Prazosin) | LRH | 82 | 100.0% | 0 | 0.0% | 82 | 100.0% |
|  | NRH | 106 | 100.0% | 0 | 0.0% | 106 | 100.0% |
|  | PA | 221 | 100.0% | 0 | 0.0% | 221 | 100.0% |
| What were the baseline antihypertensive medications? (choice=Hydralazine) | LRH | 82 | 100.0% | 0 | 0.0% | 82 | 100.0% |
|  | NRH | 106 | 100.0% | 0 | 0.0% | 106 | 100.0% |
|  | PA | 221 | 100.0% | 0 | 0.0% | 221 | 100.0% |
| What were the baseline antihypertensive medications? (choice=Methyldopa or clonidine) | LRH | 82 | 100.0% | 0 | 0.0% | 82 | 100.0% |
|  | NRH | 106 | 100.0% | 0 | 0.0% | 106 | 100.0% |
|  | PA | 221 | 100.0% | 0 | 0.0% | 221 | 100.0% |
| Systolic blood pressure (mmHg) | LRH | 76 | 92.7% | 6 | 7.3% | 82 | 100.0% |
|  | NRH | 91 | 85.8% | 15 | 14.2% | 106 | 100.0% |
|  | PA | 199 | 90.0% | 22 | 10.0% | 221 | 100.0% |
| Diastolic blood pressure (mmHg) | LRH | 76 | 92.7% | 6 | 7.3% | 82 | 100.0% |
|  | NRH | 91 | 85.8% | 15 | 14.2% | 106 | 100.0% |
|  | PA | 199 | 90.0% | 22 | 10.0% | 221 | 100.0% |
| BMI (kg/m2) | LRH | 75 | 91.5% | 7 | 8.5% | 82 | 100.0% |
|  | NRH | 103 | 97.2% | 3 | 2.8% | 106 | 100.0% |
|  | PA | 212 | 95.9% | 9 | 4.1% | 221 | 100.0% |
| Aldosterone (pmol/L) | LRH | 82 | 100.0% | 0 | 0.0% | 82 | 100.0% |
|  | NRH | 106 | 100.0% | 0 | 0.0% | 106 | 100.0% |
|  | PA | 221 | 100.0% | 0 | 0.0% | 221 | 100.0% |
| Renin (mU/L) | LRH | 82 | 100.0% | 0 | 0.0% | 82 | 100.0% |
|  | NRH | 106 | 100.0% | 0 | 0.0% | 106 | 100.0% |
|  | PA | 221 | 100.0% | 0 | 0.0% | 221 | 100.0% |
| ARR (pmol/mU) | LRH | 82 | 100.0% | 0 | 0.0% | 82 | 100.0% |
|  | NRH | 106 | 100.0% | 0 | 0.0% | 106 | 100.0% |
|  | PA | 221 | 100.0% | 0 | 0.0% | 221 | 100.0% |
| eGFR (mL/min) | LRH | 80 | 97.6% | 2 | 2.4% | 82 | 100.0% |
|  | NRH | 104 | 98.1% | 2 | 1.9% | 106 | 100.0% |
|  | PA | 218 | 98.6% | 3 | 1.4% | 221 | 100.0% |
| Sodium (mmol/L) | LRH | 80 | 97.6% | 2 | 2.4% | 82 | 100.0% |
|  | NRH | 105 | 99.1% | 1 | 0.9% | 106 | 100.0% |
|  | PA | 219 | 99.1% | 2 | 0.9% | 221 | 100.0% |
| Potassium (mmol/L) | LRH | 80 | 97.6% | 2 | 2.4% | 82 | 100.0% |
|  | NRH | 105 | 99.1% | 1 | 0.9% | 106 | 100.0% |
|  | PA | 219 | 99.1% | 2 | 0.9% | 221 | 100.0% |
| Bicarbonate (mmol/L) | LRH | 78 | 95.1% | 4 | 4.9% | 82 | 100.0% |
|  | NRH | 105 | 99.1% | 1 | 0.9% | 106 | 100.0% |
|  | PA | 214 | 96.8% | 7 | 3.2% | 221 | 100.0% |
| Creatinine (umol/L) | LRH | 79 | 96.3% | 3 | 3.7% | 82 | 100.0% |
|  | NRH | 105 | 99.1% | 1 | 0.9% | 106 | 100.0% |
|  | PA | 216 | 97.7% | 5 | 2.3% | 221 | 100.0% |
| Urine Albumin: creatinine ratio (mg/mmol) | LRH | 40 | 48.8% | 42 | 51.2% | 82 | 100.0% |
|  | NRH | 63 | 59.4% | 43 | 40.6% | 106 | 100.0% |
|  | PA | 95 | 43.0% | 126 | 57.0% | 221 | 100.0% |
| Glucose (fasting, mmol/L) | LRH | 48 | 58.5% | 34 | 41.5% | 82 | 100.0% |
|  | NRH | 76 | 71.7% | 30 | 28.3% | 106 | 100.0% |
|  | PA | 82 | 37.1% | 139 | 62.9% | 221 | 100.0% |
| Insulin (fasting, mU/L) | LRH | 23 | 28.0% | 59 | 72.0% | 82 | 100.0% |
|  | NRH | 50 | 47.2% | 56 | 52.8% | 106 | 100.0% |
|  | PA | 41 | 18.6% | 180 | 81.4% | 221 | 100.0% |
| Total cholesterol (fasting, mmol/L) | LRH | 52 | 63.4% | 30 | 36.6% | 82 | 100.0% |
|  | NRH | 78 | 73.6% | 28 | 26.4% | 106 | 100.0% |
|  | PA | 99 | 44.8% | 122 | 55.2% | 221 | 100.0% |
| LDL (fasting, mmol/L) | LRH | 48 | 58.5% | 34 | 41.5% | 82 | 100.0% |
|  | NRH | 73 | 68.9% | 33 | 31.1% | 106 | 100.0% |
|  | PA | 89 | 40.3% | 132 | 59.7% | 221 | 100.0% |
| HDL (fasting, mmol/L) | LRH | 48 | 58.5% | 34 | 41.5% | 82 | 100.0% |
|  | NRH | 76 | 71.7% | 30 | 28.3% | 106 | 100.0% |
|  | PA | 90 | 40.7% | 131 | 59.3% | 221 | 100.0% |
| Triglyceride (fasting, mmol/L) | LRH | 52 | 63.4% | 30 | 36.6% | 82 | 100.0% |
|  | NRH | 77 | 72.6% | 29 | 27.4% | 106 | 100.0% |
|  | PA | 98 | 44.3% | 123 | 55.7% | 221 | 100.0% |
| Urine volume (mL) | LRH | 45 | 54.9% | 37 | 45.1% | 82 | 100.0% |
|  | NRH | 64 | 60.4% | 42 | 39.6% | 106 | 100.0% |
|  | PA | 113 | 51.1% | 108 | 48.9% | 221 | 100.0% |
| Sodium excretion (mmol/day) | LRH | 41 | 50.0% | 41 | 50.0% | 82 | 100.0% |
|  | NRH | 57 | 53.8% | 49 | 46.2% | 106 | 100.0% |
|  | PA | 99 | 44.8% | 122 | 55.2% | 221 | 100.0% |
| Potassium excretion (mmol/day) | LRH | 42 | 51.2% | 40 | 48.8% | 82 | 100.0% |
|  | NRH | 55 | 51.9% | 51 | 48.1% | 106 | 100.0% |
|  | PA | 98 | 44.3% | 123 | 55.7% | 221 | 100.0% |
| Aldosterone excretion (nmol/day) | LRH | 33 | 40.2% | 49 | 59.8% | 82 | 100.0% |
|  | NRH | 45 | 42.5% | 61 | 57.5% | 106 | 100.0% |
|  | PA | 99 | 44.8% | 122 | 55.2% | 221 | 100.0% |
| Pre-SST: Potassium (supine, mmol/L) | LRH | 2 | 2.4% | 80 | 97.6% | 82 | 100.0% |
|  | PA | 74 | 33.5% | 147 | 66.5% | 221 | 100.0% |
| Pre-SST: Aldosterone (supine, pmol/L) | LRH | 3 | 3.7% | 79 | 96.3% | 82 | 100.0% |
|  | PA | 81 | 36.7% | 140 | 63.3% | 221 | 100.0% |
| Pre-SST: Cortisol (supine, nmol/L) | LRH | 3 | 3.7% | 79 | 96.3% | 82 | 100.0% |
|  | PA | 77 | 34.8% | 144 | 65.2% | 221 | 100.0% |
| Pre-SST: Renin (supine, mU/L) | LRH | 3 | 3.7% | 79 | 96.3% | 82 | 100.0% |
|  | PA | 81 | 36.7% | 140 | 63.3% | 221 | 100.0% |
| Pre-SST: ARR (supine, pmol/mU) | LRH | 3 | 3.7% | 79 | 96.3% | 82 | 100.0% |
|  | PA | 77 | 34.8% | 144 | 65.2% | 221 | 100.0% |
| Post-SST: Potassium (supine, mmol/L) | LRH | 3 | 3.7% | 79 | 96.3% | 82 | 100.0% |
|  | PA | 77 | 34.8% | 144 | 65.2% | 221 | 100.0% |
| Post-SST: Aldosterone (supine, pmol/L) | LRH | 3 | 3.7% | 79 | 96.3% | 82 | 100.0% |
|  | PA | 82 | 37.1% | 139 | 62.9% | 221 | 100.0% |
| Post-SST: Cortisol (supine, nmol/L) | LRH | 3 | 3.7% | 79 | 96.3% | 82 | 100.0% |
|  | PA | 79 | 35.7% | 142 | 64.3% | 221 | 100.0% |
| Post-SST: Renin (supine, mU/L) | LRH | 3 | 3.7% | 79 | 96.3% | 82 | 100.0% |
|  | PA | 80 | 36.2% | 141 | 63.8% | 221 | 100.0% |
| Post-SST: ARR (supine, pmol/mU) | LRH | 2 | 2.4% | 80 | 97.6% | 82 | 100.0% |
|  | PA | 73 | 33.0% | 148 | 67.0% | 221 | 100.0% |
| Pre-SST: Potassium (Seated, mmol/L) | LRH | 31 | 37.8% | 51 | 62.2% | 82 | 100.0% |
|  | NRH | 6 | 5.7% | 100 | 94.3% | 106 | 100.0% |
|  | PA | 130 | 58.8% | 91 | 41.2% | 221 | 100.0% |
| Pre-SST: Aldosterone (seated, pmol/L) | LRH | 31 | 37.8% | 51 | 62.2% | 82 | 100.0% |
|  | NRH | 6 | 5.7% | 100 | 94.3% | 106 | 100.0% |
|  | PA | 135 | 61.1% | 86 | 38.9% | 221 | 100.0% |
| Pre-SST: Cortisol (seated, nmol/L) | LRH | 31 | 37.8% | 51 | 62.2% | 82 | 100.0% |
|  | NRH | 6 | 5.7% | 100 | 94.3% | 106 | 100.0% |
|  | PA | 131 | 59.3% | 90 | 40.7% | 221 | 100.0% |
| Pre-SST: Renin (seated, mU/L) | LRH | 31 | 37.8% | 51 | 62.2% | 82 | 100.0% |
|  | NRH | 6 | 5.7% | 100 | 94.3% | 106 | 100.0% |
|  | PA | 134 | 60.6% | 87 | 39.4% | 221 | 100.0% |
| Pre-SST: ARR (seated, pmol/mU) | LRH | 30 | 36.6% | 52 | 63.4% | 82 | 100.0% |
|  | NRH | 6 | 5.7% | 100 | 94.3% | 106 | 100.0% |
|  | PA | 121 | 54.8% | 100 | 45.2% | 221 | 100.0% |
| Post-SST: Aldosterone (seated, pmol/L) | LRH | 31 | 37.8% | 51 | 62.2% | 82 | 100.0% |
|  | NRH | 6 | 5.7% | 100 | 94.3% | 106 | 100.0% |
|  | PA | 135 | 61.1% | 86 | 38.9% | 221 | 100.0% |
| Post-SST: Potassium (seated, mmol/L) | LRH | 31 | 37.8% | 51 | 62.2% | 82 | 100.0% |
|  | NRH | 6 | 5.7% | 100 | 94.3% | 106 | 100.0% |
|  | PA | 129 | 58.4% | 92 | 41.6% | 221 | 100.0% |
| Post-SST: Cortisol (seated, nmol/L) | LRH | 31 | 37.8% | 51 | 62.2% | 82 | 100.0% |
|  | NRH | 5 | 4.7% | 101 | 95.3% | 106 | 100.0% |
|  | PA | 131 | 59.3% | 90 | 40.7% | 221 | 100.0% |
| Post-SST: Renin (seated, mU/L) | LRH | 30 | 36.6% | 52 | 63.4% | 82 | 100.0% |
|  | NRH | 6 | 5.7% | 100 | 94.3% | 106 | 100.0% |
|  | PA | 133 | 60.2% | 88 | 39.8% | 221 | 100.0% |
| Post-SST: ARR (seated, pmol/mU) | LRH | 28 | 34.1% | 54 | 65.9% | 82 | 100.0% |
|  | NRH | 6 | 5.7% | 100 | 94.3% | 106 | 100.0% |
|  | PA | 115 | 52.0% | 106 | 48.0% | 221 | 100.0% |

ACE: angiotensin converting enzyme; ARB: angiotensin II receptor blocker; ARR: aldosterone-to-renin ratio, BMI: body mass index; eGFR: estimated glomerular filtration rate; HDL: high-density lipoprotein; low-density lipoprotein; LRH: low-renin hypertension; MRA: mineralocorticoid receptor antagonist; N: number; NRH: normal-renin hypertension; PA: primary aldosteronism; SST: saline suppression test.


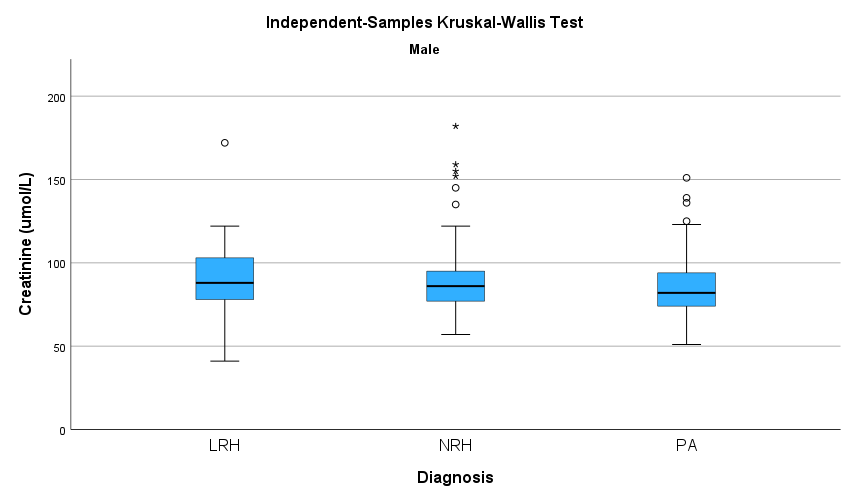


Figure S1. Box and whisker plots of serum creatinine in male patients.

LRH: low-renin hypertension; NRH: normal-renin hypertension; PA: primary aldosteronism.


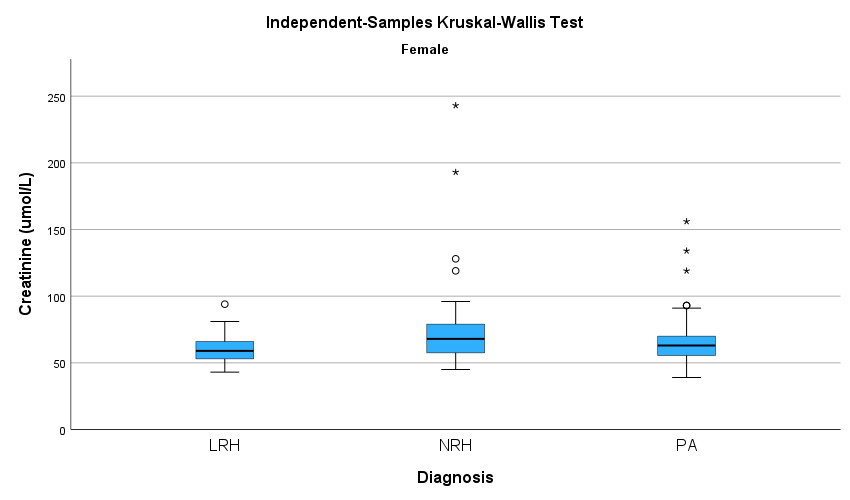


Figure S2. Box and whisker plots of serum creatinine in female patients.

LRH: low-renin hypertension; NRH: normal-renin hypertension; PA: primary aldosteronism.
